# Structural insight into interleukin‐4Rα and interleukin‐5 inhibition by nanobodies from a bispecific antibody

**DOI:** 10.1002/mco2.700

**Published:** 2024-09-09

**Authors:** Weicheng Qiu, Jinguo Meng, Zhipeng Su, Wei Xie, Gaojie Song

**Affiliations:** ^1^ Shanghai Key Laboratory of Regulatory Biology Institute of Biomedical Sciences and School of Life Sciences East China Normal University Shanghai China; ^2^ The Thoth Institutes for Health Research, RegeneCore Biotech Co., Ltd Nanjing China; ^3^ The Key Laboratory of Developmental Genes and Human Disease, Ministry of Education and The School of Life Science and Technology Southeast University Nanjing China

Dear Editor,

Specific immunological challenges can stimulate T helper 2 (Th2) cells and trigger immune response by secreting cytokines such as interleukin‐4 (IL‐4), IL‐5, and IL‐13, and dysregulation of these cytokines are closely related to the pathogenesis of diseases such as atopic dermatitis and allergic asthma.^1^ At the molecular level, these cytokines function by binding to different cytokine receptors, thus triggering various inflammatory responses. IL‐4 can either bind individually via a heterodimeric receptor composed of IL‐4Rα(CD124) and γc (CD132) to trigger a type I inflammatory response or signal through a shared type II inflammatory response with IL‐13 to bind another heterodimeric receptor composed of IL‐4Rα and IL‐13α1. Both types of receptor complexes can activate the phosphorylation of the signal transducer and activator of the transcription (STAT6) pathway via Janus kinase. In addition, IL‐5 functions via the IL‐5 receptor, which is also a heterodimer consisting of a specific α subunit for binding (IL‐5Rα) and a shared β subunit for signal transduction (colony‐stimulating factor 2 receptor beta, CSF2RB). Currently, several monoclonal antibody drugs are approved for marketing worldwide for asthma indications, but these are mainly single‐target blockers related to IL‐4, IL‐5, and IL‐13 signaling pathways,^1^ and most of these medicines have individual limitations. According to investigations of Th2 cell‐related signaling pathways and the outcomes of some clinical trials,^2^ blocking different steps within the type II inflammatory pathway may produce better efficacy as this approach is expected to produce synergistic effects.

Here, we attempted to develop a bispecific antibody targeting both IL‐4Rα and IL‐5 within the type II inflammatory pathway. To obtain the VHHs, we immunized alpaca with individual proteins of IL‐4Rα or IL‐5 (Figure 1A). Taking IL‐4Rα for example, total RNA was extracted from lymphocytes to construct a VHH library. 109 unique binders for IL4Rα were identified after library screening. After validating binding and blocking capacity, we selected two binders (dAb1 and dAb2) for further engineering and humanization. These two nanobodies exhibited similar EC_50_ (single digit nanomolar) when associated with IL‐4Rα in either VHH or Fc‐fused form, with dAb1 performing slightly better when blocking the IL‐4**–**IL‐4Rα interaction (Figure S1A–D). We further found that dAb1 shows an IC_50_ of ∼0.15 nM in either IL‐4 or IL‐13‐induced TF‐1 cell proliferation assay (Figure 1B), which is comparable with the efficacy of dupilumab (Figure S1E,F). Subsequently, dAb1 was selected and recombined with the best VHH against IL‐5 (which was generated and selected using a similar process) to produce a bispecific antibody.

**FIGURE 1:**
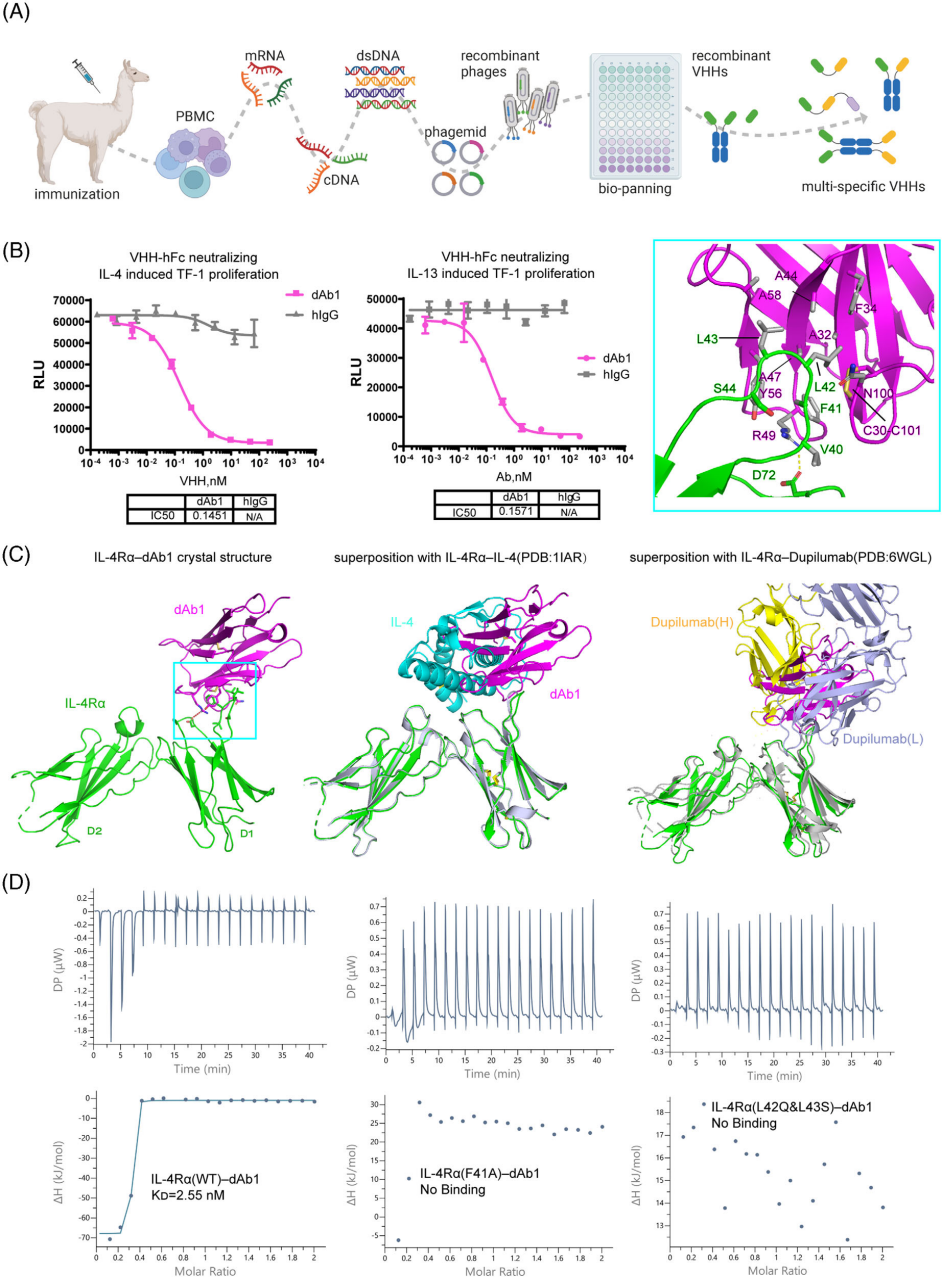
Development and characterization of neutralizing VHHs against interleukin (IL)‐4Rα. (A) Schematic diagram of workflow for VHH generation. (B) dAb1 blocked the IL‐4 (left) or IL‐13 (right) induced TF‐1 cell proliferation. hIgG was used as a negative control in the cell proliferation assay. (C) Crystal structure of IL‐4Rα in complex with dAb1(left) and comparison with the IL‐4–IL‐4Rα (middle) or Dupilumab–IL‐4Rα(right). Proteins are colored‐coded. The detailed interaction between IL‐4Rα and dAb1 is shown in the pane of the top right. Key interacting residues are shown as sticks and labeled. (D) Isothermal titration calorimetry (ITC) measurement of the affinity of dAb1 with wild‐type (WT) or mutated IL‐4Rα.

Interestingly, we found the IL4Rα‐binding nanobody dAb1 binds only to human IL‐4Rα but has no cross‐reactivity with IL‐4Rα from macaque or mouse (Figure S1G), whereas the IL‐5‐binding nanobody can bind to macaque IL‐5. To further gain insights into the molecular mechanism of the blocking capability and binding specialization, we tried to co‐crystallize the individual VHH with each corresponding antigen. We were able to determine the crystal structure of human IL4Rα in complex with dAb1 to a moderate resolution (3.96 Å). we used previous high‐resolution structures to model the fibronectin type III domains from IL‐4Rα (named D1 and D2 from N‐terminus to C‐terminus) and the immunoglobulin domain of dAb1 (Figure 1C and Table S1).^3^, ^4^ The disulfide bonds within IL‐4Rα and dAb1, the N‐glycosylation sites, as well as the residues within the binding interface were well modeled based on the electron densities (Figure S1H). Compared to the physiological ligand IL‐4 which binds to the conjunction region between D1 and D2 (Figure 1C), our dAb1 binds solely to the V^40^FLLS^44^ loop region within the D1 domain. The superposition of the two structures shows that the binding of dAb1 can preclude its association with IL‐4, explaining the mechanism of action for dAb1's blocking of the IL‐4–IL‐4Rα interaction for immune regulation (Figure 1C, middle). Since IL‐13 also binds to the same region on IL‐4Rα as IL‐4, it is readily concluded that dAb1 should also block the association of IL‐13 and IL‐4Rα. Similarly, the previously approved anti‐IL‐4Rα Fab, Dupilumab, binds mainly to the same loop region within the D1 domain (Figure 1C, right).^3^ Dupilumab binds with a larger interface (1056 Å^2^) compared to the nanobody dAb1 (625 Å^2^), consistent with its stronger binding affinity (33 pM) with IL‐4Rα as previously reported.^5^

The V^40^FLLS^44^ loop of D1 binds mainly through nonpolar interactions with a hydrophobic patch on one side of dAb1 (Figure 1C, embedded). Specifically, F41 of D1 forms a π‐cation interaction with R49 and an edge‐to‐face π‐π interaction with Y56; L42 and L43 of D1 are surrounded by hydrophobic residues A32, F34, A44, A47, Y56 and A58. In addition to these nonpolar interactions, R49 of dAb1 also forms a salt bridge with D72 of IL‐4Rα. The V^40^FLLS^44^ loop is highly varied within IL4Rα of different species (Figure S1I). L42 and L43 of human IL4Rα are replaced by polar residues or bulky side‐chain residues in other species, thus explaining the binding specificity between dAb1 and human IL‐4Rα. To further validate the visualized interface in the crystal structure, we made several mutations to IL‐4Rα and measured the affinity with dAb1 by isothermal titration calorimetry (ITC). The results showed that dAb1 can bind to human IL‐4Rα with an affinity of single‐digit nanomolar, which is similar to the affinity between IL‐4 and IL‐4Rα.^4^ In contrast, the F41A mutant, as well as the macaque‐mimicking double mutant (L42Q, L43S), no longer bind to dAb1 (Figure 1D). These results further confirmed the observed interface and explained the selectivity of dAb1 on human IL‐4Rα from a molecular level.

Since we were not able to crystallize the IL‐5 and its cognate VHH, the recently released AlphaFold3 server was used to generate potential models for the complex. The ITC titration suggested two binding sites of our VHH on the dimeric IL‐5, with an affinity of ∼7 nM (Figure S2A). The negligible effects on VHH‐binding by the tested IL‐5 mutations of D117, T128, and E129 favored a complex model in which the VHHs bind symmetrically to the cleft between the dimeric interface of IL‐5, a region that is also the key place recognized by the fibronectin domains of IL‐5Rα (Figure S2B,C). Thus, these data indicate that the VHH against IL‐5 breaks the interaction between IL‐5 and IL‐5Rα, laying a foundation for the physiological function of the bispecific antibody.

In summary, we developed a bispecific antibody that targets both IL‐4Rα and IL‐5 from humans using humanized VHHs derived from alpaca, and studied the epitope of our VHHs on IL‐4Rα and IL‐5. The structural and biochemical data explained the inhibition mechanism of our nanobodies on the interactions between IL‐4, IL‐5, IL‐13, and their receptors. Dupilumab has shown robust clinical efficacy on type 2 diseases by targeting IL‐4Rα, the shared receptor of IL‐4 and IL‐13. Nevertheless, dupilumab performs relatively poorly in patients with severe disease. Notably, our bispecific antibody targeting two proteins (IL‐5 and IL‐4Rα) is expected to have promising efficacy as it can simultaneously attenuate the function of three cytokines (IL‐4, IL‐5, and IL‐13). Although there are still challenges for bispecific antibodies, like off‐target toxicity or insufficient co‐stimulation, this strategy has become more and more popular and many clinical trials are currently underway to satisfy different clinical demands.

## AUTHOR CONTRIBUTIONS

W.X. and G.S. conceived the project; W.Q. optimized constructs for dAb1 and IL‐4Rα, expressed and purified the complex proteins for crystallization, conducted mutagenesis, and edited the initial manuscript; J.M. conducted animal immunization, antibody recombination and purification; Z.S. designed and carried out the ELISA and cell assays for VHH validation, and edited the manuscript; W.X. and G.S. supervised the project, and wrote the manuscript. All authors were involved in the discussion and provided feedback for the manuscript.

## CONFLICT OF INTEREST STATEMENT

Su Zhipeng and Meng Jinguo are employees of RegeneCore Biotech Co., Ltd. The other authors declare no conflict of interest.

## ETHICS STATEMENT

Not applicable.

## Supporting information

Supporting Information

## Data Availability

The atomic coordinate and structure factor for the IL‐4Rα–dAb1 complex has been deposited in the Protein Data Bank with identification code 8Z8L. Correspondence and requests for materials should be addressed to wei.xie@seu.edu.cn or gjsong@bio.ecnu.edu.cn
