# Supplementary material for: Structural insight into interleukin‐4Rα and interleukin‐5 inhibition by nanobodies from a bispecific antibody
Source: MedComm (2020). 2024 Sep 9;5(9):e700. doi: 10.1002/mco2.700 (PMC11381654; doi:10.1002/mco2.700)
Supplement: Supplementary file 1 — Supporting Information [file MCO2-5-e700-s001.docx]

Supplemental Materials

**Structural insight into IL-4Rα and IL-5 inhibition by nanobodies from a bispecific antibody**

Weicheng Qiu^1^, Jinguo Meng^2^, Zhipeng Su^2^, Wei Xie^3,^ *, and Gaojie Song^1,^ *

^1^ Shanghai Key Laboratory of Regulatory Biology, Institute of Biomedical Sciences and School of Life Sciences, East China Normal University, 500 Dongchuan Road, Shanghai 200241, China.

^2^ The Thoth Institutes for Health Research; RegeneCore Biotech Co., Ltd, Building 7A, Treehouse Campus, 73 Tanmi Road, Nanjing, 210031, China.

^3^ The Key Laboratory of Developmental Genes and Human Disease, Ministry of Education, The School of Life Science and Technology, Southeast University, 2 Dongda Road, Nanjing, 210032, China

Correspondence: [wei.xie@seu.edu.cn](mailto:wei.xie@seu.edu.cn) or gjsong@bio.ecnu.edu.cn

**Methods**

**Alpaca immunization and antibody screening**

For generation of target specific VHH, alpaca was immunized with recombinant antigens (produced by Regenecore) for four subcutaneous injections in 2-weeks interval. The peripheral blood was collected after last immunization to construct VHHs library. Total RNA was extracted from peripheral blood lymphocyte isolated with Ficoll-Paque PLUS (17-1440-03; GE), and subjected to reverse transcription to cDNA via SuperScript III reverse transcriptase (18080093; Life Tech). The synthesized cDNA was used as template in PCR to amplify the VHH encoding sequences. The VHHs repertoire were recombinated into pMECS phagemid (Biovector501740; BioVector NTCC Inc.), and the recombinants were electro-transformed into TG1 cells. Capacity and diversity of the library were analyzed by serial dilution and individual colony sequencing. For initial selections, 500 ng target protein in 100 μL coating buffer (100 mmol/L NaHCO_3_, pH 8.4) was coated overnight on the Maxisorp 96-well Immunoplates (439454; Nunc) at 4°C, and 2% skimmed milk in PBS was used as blocking buffer. Subsequently, the recombinational phages rescued from VHHs library were incubated with antigen-coated well (and control) at room temperature for 1 hour. Excessive phages were washed by PBST and bound phages were eluted by 100 μL of 250 μg/mL trypsin/PBS. To calculate the enrichment parameter, the eluted phages were serial diluted and then used to infect TG1 cells. After 1 hour, the infected TG1 cells were plated on LB agar plates supplied with glucose and ampicillin. For periplasmic extract ELISA, 94 individual colonies were selected randomly. Right after that, colonies were cultured and induced by isopropyl β-d-1-thiogalactopyranoside (IPTG) for VHHs’ production. The binding ability of extracted VHHs with antigen were tested by ELISA individually, and positive colonies were sequenced. Finally, the selected clones were subcloned into pET28a vector and transformed into BL-21 (DE3) *E. coli* cells for large scale expression and purified via immobilized metal affinity chromatography (IMAC). All the above was completely achieved by Regenecore.

**Antibody identification and recombination**

The codon-optimized antibody coding sequences were synthesized into the vector pcDNA3.4 by sequence synthesis, and then the vectors were transfected into suspension 293 cells for expression, and the recombinant antibodies were purified by protein A resin.

For the receptor binding assay, the ligand protein conjugated to biotin and the purified recombinant antibody were each diluted to the appropriate concentration, and then the two proteins were mixed in a 1:1 volume ratio. The mixture binds to the receptor protein coated on the plate by ELISA. Finally, the signal of biotin is determined. In this way, the dose-response relationship of the antibody blocking receptor and ligand binding effect is obtained. All the above was completely achieved by Regenecore.

**Cell proliferation assay**

TF-1 cells were seeded with a density of 2×10^5^ cells per ml in RPMI 1640 with 10% FBS. The recombinant VHHs were diluted to 40 μg/mL, from which 5-fold serial dilutions were set: 40 μg/ml, 8 μg/mL, 1.6 μg/mL, 0.32 μg/mL, 0.064 μg/mL, 0.0128 μg/mL, 0.00256 μg/mL, 0.0.000512 μg/mL, 0.0001024 μg/mL, and 0 μg/mL. Mix IL-4 or IL-13 with the antibody solution at different concentrations, then add 50 μL mixture to each well seeded with TF-1 cells, two replicates were carried out. After incubation for 72 h, detect luminescence of each well after adding CellTiter-Glo.

**Protein purification and complex formation**

The dAb1 and IL-4Rα (WT) were each cloned into pcDNA3.4 vector and expressed in HEK293S GnTI^-^ cells. Protein containing cell culture supernatants were harvested and clarified media was purified by Ni Smart Beads 6FF (Smart-Lifesciences) using PBS buffer plus 15 mM imidazole, pH 7.5 as the binding buffer. The proteins were then eluted with a 10-column volume of PBS plus 0.3 M imidazole, pH 7.4. The eluents were collected and concentrated using a Millipore 10 kDa spin concentrator. The concentrated proteins were loaded on a Superdex75 (Uniondex) columns. Proteins were concentrated again to 10 mg/mL for complex formation. IL-4Rα (WT) and dAb1 were mixed at molar ratio 1:1.5 and incubated at 4℃, overnight. The complex was characterized by analytical size exclusion chromatography and SDS-PAGE. The IL-4Rα mutants (F40A, L41Q&L42S) were expressed and purified similarly and used together with WT proteins for ITC measurements. IL-5 and its mutants were also cloned and expressed from HEK293S GnTI^-^ cells and purified similarly.

**Crystallization and structure determination**

The complex samples were concentrated to 22 mg/mL and were set up at room temperature in vapor diffusion hanging drops at a volume ratio of 1:1 using Molecular Dimensions Proplex, MIDASplus and Hampton PEG, Natrix, Crystal Screen crystallization screens. Images of crystallization trays were taken on day 3, day 7, day 10 and day 20. The crystals were generated in a mother solution of 1.4 M sodium malonate dibasic monohydrate pH 6.0 at 16℃. Prior to freezing in liquid nitrogen, crystals were transferred to a cryoprotectant solution composed of the mother solution with 10% glycerol. Structure determination diffraction datasets were collected at beamline BL10U2 of Shanghai Synchrotron Radiation Facility (SSRF). Data were integrated and reduced using XDS package.^1^ Initial molecular replacement solutions were obtained using Phaser (CCP4 suite).^2^ The model was built using COOT^3^ and refined by Phenix.^4^ Structure-related figures were generated using Pymol (https://www.pymol.org). The structure-based sequence alignment was conducted using CLUSTALW and presented by ESPript server.^5^

**Isothermal titration calorimetry**

The Isothermal Titration Calorimetry (ITC) experiments were performed using a MicroCal PEAQ-ITC instrument (Malvern) at a temperature of 25 °C. The protein samples were dissolved in 20 mM HEPES pH 7.5 and 150 mM NaCl, with IL4Rα used as the fixed sample and dAb1 as the titration sample. To measure the binding affinity between dAb1 and IL-4Rα WT or mutants, 40 μl of dAb1 (200 μM) in the syringe was titrated into 300 μl of IL-4Rα (20 μM) samples in the cell. Data were analyzed using MicroCal PEAQ-ITC Analysis Software. ITC measurement between IL-5 and its cognate VHH was also conducted and analyzed similarly.

1. Kabsch, W. Xds. *Acta Crystallographica Section D Biological Crystallography* **66**, 125-132 (2010).
2. McCoy, A.J. et al. Phasercrystallographic software. *Journal of Applied Crystallography* **40**, 658-674 (2007).
3. Emsley, P., Lohkamp, B., Scott, W.G. & Cowtan, K. Features and development of Coot. *Acta Crystallographica Section D Biological Crystallography* **66**, 486-501 (2010).
4. Adams, P.D. et al. PHENIX: a comprehensive Python-based system for macromolecular structure solution. *Acta Crystallographica Section D Biological Crystallography* **66**, 213-221 (2010).
5. Robert, X. & Gouet, P. Deciphering key features in protein structures with the new ENDscript server. *Nucleic Acids Research* **42**, W320-W324 (2014).

**Supplemental Figures**

**Figure S1. Development and characterization of VHHs against IL-4Rα.** A-B. Binding (A-B) and Blocking efficacy (C-D) of selected nanobodies on human IL-4Rα with VHH or human Fc-fused form. E-F. dupilumab blocked the IL-4 (E) or IL-13 (F) induced TF-1 cell proliferation. hIgG was used as negative control in the asssay. G. ELISA measurement of different species of IL-4Rα with dAb1-hFc. H. Typical densities (σ=1) for the crystal structure solved in current study. I. Sequence alignment of IL-4Rα from different species. The conserved residues are shown with red background, and the key varied residues are highlighted with green pane.


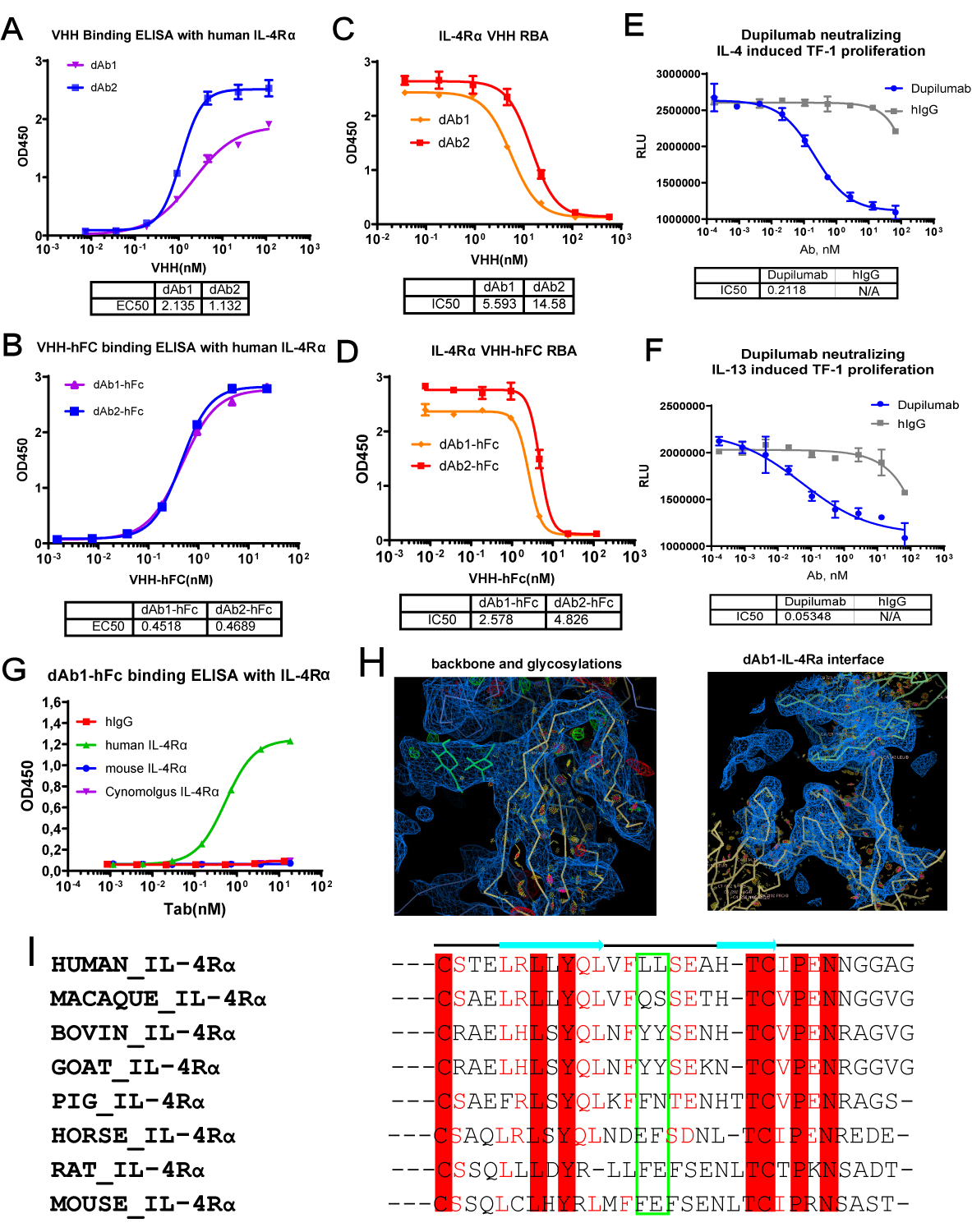


**Figure S2. Characterization of VHH against IL-5.** A. ITC measurement of the affinity between IL-5 and its cognate VHH. B. Co-migration of the IL-5 and its mutants with the cognate VHH from gel filtration. C. Binding model selected from the predicted AlphaFold3 server and superposition with the IL-5–IL-5Rα complex.

**
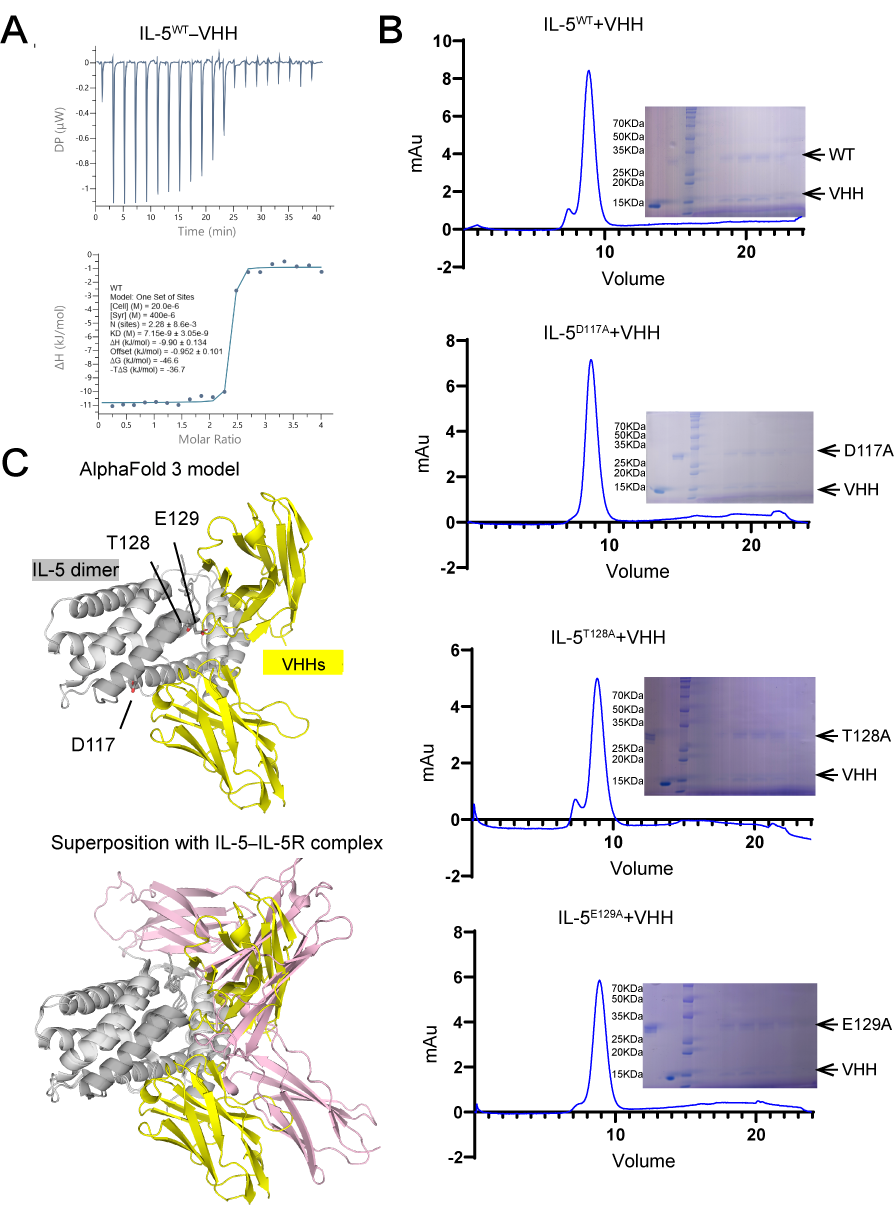
**

**Table S1. data collection and refinement statistics**

|  | **IL-4Rα–dAb1** |
| --- | --- |
| **Data collection** |  |
| Space group | H32 |
| Cell dimensions |  |
| *a*, *b*, *c* (Å) | 142.94, 142.94, 149.08 |
| α, β, γ (°) | 90.00, 90.00, 120.00 |
| Resolution (Å)^a^ | 95.24-3.96 (4.21-3.96) |
| Reflections (total/unique) | 56100/4534 |
| *R*_merge_^b^ | 0.31 (4.94) |
| CC1/2^c^ | 0.99(0.38) |
| I/σ(I) | 7.2 (1.3) |
| Completeness (%) | 92.1 (42.5) |
| Redundancy | 12.4(13.0) |
| **Refinement** |  |
| No. reflections | 4488 |
| *R*_work/_ *R*_free_ | 0.301/0.334 |
| R.m.s deviations |  |
| Bond lengths (Å) | 0.003 |
| Bond angles (º) | 0.672 |
| Ramachandran (%)^d^ | 89.8/9.9/0.3 |
| PDB ID |  |

^a^Values for highest resolution shells are given in parentheses.

^b^Rmerge = ∑*hkl*∑_i_*|*I_i_(*hkl*) − <I(*hkl*)*>|*/∑*hkl*∑_i_*|*I_i_(*hkl*) where I_i_ (*hkl*) and <I(*hkl*)> are the i and mean measurement of intensity of reflection *hkl.*^c^CC1/2 = Pearson’s correlation coefficient between average intensities of random half data sets for each unique reflection.

^d^Residues in favored, accepted, and outlier regions of the Ramachandran plot as reported by MOLPROBITY
